# Right versus left eye asymmetry of microvasculature in diabetes revealed by optical coherence tomography angiography

**DOI:** 10.1038/s41598-023-36058-8

**Published:** 2023-06-08

**Authors:** Tong Zhao, Sawarin Laotaweerungsawat, Yi Chen, Xiuyun Liu, Dongwei Liu, Jay M. Stewart

**Affiliations:** 1 Department of Ophthalmology, University of California, San Francisco, San Francisco, CA USA; 2 Department of Ophthalmology, Zuckerberg San Francisco General Hospital and Trauma Center, San Francisco, CA USA; 3 Department of Ophthalmology, China-Japan Friendship Hospital, Beijing, China; 4 Department of Ophthalmology, Charoenkrung Pracharak Hospital, Bangkok, Thailand; 5 Shenzhen Key Laboratory of Ophthalmology, Shenzhen Eye Hospital, Jinan University, Shenzhen, China; 6 School of Optometry, Shenzhen University, Shenzhen, China; 7 Department of Physiological Nursing, University of California, San Francisco, San Francisco, CA USA; 8 Department of Ophthalmology, The Second Affiliated Hospital of Anhui Medical University, Hefei, China

**Keywords:** Retinal diseases, Diabetes complications

## Abstract

In this study, we explored inter-ocular asymmetry (between the two eyes of the same patient) using optical coherence tomography angiography (OCTA) in patients with diabetes mellitus (DM) at different retinopathy stages. A total of 258 patients were divided into four groups: no DM, DM without diabetic retinopathy (DR), non-proliferative DR (NPDR), and proliferative DR (PDR). Superficial and deep vessel density (SVD, DVD), superficial and deep perfusion density (SPD, DPD), foveal avascular zone (FAZ) area, perimeter and circularity were calculated, and asymmetry index (AI) was used to evaluate the asymmetry of two eyes of the same subject. AIs of SPD, SVD, FAZ area and FAZ perimeter in the PDR group were larger than all other 3 groups (all *p* < 0.05). The AIs of DPD, DVD, FAZ area and FAZ perimeter in males were larger than in females (*p* = 0.015, *p* = 0.023, *p* = 0.006 and *p* = 0.017). Hemoglobin A1c (HbA1c) was positively correlated with AI of FAZ perimeter (*p* = 0.02) and circularity (*p* = 0.022). In conclusion, PDR patients’ eyes were significantly asymmetric in both vascular density and FAZ metrics. Male sex and HbA1c are risk factors that influenced symmetry. This study highlights that right-left asymmetry should be taken into account in DR-related studies, particularly those analyzing microvascular changes with OCTA.

## Introduction

Diabetic retinopathy (DR) is one of the major causes of blindness worldwide and has usually been considered to be symmetric between a patient's right and left eyes based on examinations and photos^1,2^. Asymmetric DR has been reported, usually in cases with proliferative DR in one eye and nonproliferative DR in the other^3^. Some of the asymmetry cases were related to the effects of a unilateral vasculopathy, such as unilateral branch retinal vein occlusion and stenosis of the internal carotid artery or ophthalmic artery, known as ocular ischemic syndrome^4,5^. Other factors that may lead to aggravated DR in one eye include partial posterior vitreous detachment and cataract surgery, while complete posterior vitreous detachment, optic atrophy, retinitis pigmentosa, elongation of axial length, healed choroiditis and vitrectomy were believed to be protective. Uveitis and glaucoma may also cause asymmetric DR, but the significance of these factors is controversial^6–8^. In this study we focused on eyes that presented as symmetric clinically, and we sought to investigate whether the symmetry of retinopathy could be confirmed on evaluation of the density of the microvasculature. We also studied whether the retinopathy in a patient’s two eyes tended to be more asymmetric as DR progressed to higher degrees of severity. Understanding whether DR affects a patient's eyes symmetrically is important for planning clinical studies and measuring the effects of treatment interventions. If the microvascular changes of DR in the macula are not symmetric, the random selection of the enrolled eye in a research protocol may not represent the two eyes collectively. In addition, finding differences between the two eyes in the microvasculature could help predict which eye will develop complications first and could contribute toward identifying early features of unilateral systemic or ocular diseases.

Optical coherence tomography angiography (OCTA) technology, which provides noninvasive assessments of the retinal microvasculature, has been widely used in both clinical practice and research. Multiple quantitative parameters relating to the macular microvasculature can be acquired using OCTA and have been found to be useful in assessing the severity of DR^8–11^. However, little attention has been paid to the symmetry of the two eyes within the same patient. In the present study, OCTA was used to evaluate the asymmetry between the two eyes in patients with DR at different stages; we also aimed to identify potential systemic and ocular factors that might be associated with the asymmetry.

## Results

### Demographic and clinical characteristics of study subjects

A total of 258 patients were included in the study. The mean age was 52 years old, ranging from 32 to 83, with 124 females (48.1%). The distribution of race and ethnicity was as follows: non-Hispanic white 11.6%, non-Hispanic black 13.2%, Hispanic 47.3%, Asian 17.8%, others 5.2% and unspecified 8.1%. Of all subjects, 52% had a history of hypertension, 56% had hyperlipidemia. 43% were obese, and 19% had a history of smoking. The mean axial length was 23.55 ± 0.96 mm. The mean CMT was 239 ± 20 μm. There were 60 patients in the control/no DM group, 132 in the DM without DR group, 40 with NPDR and 26 with PDR. Of the 258 subjects, only one patient had different DR stages between the two eyes and was placed into the PDR group; in that patient, one eye was graded as PDR, while the other was severe NPDR. There were no significant differences between the four groups concerning age, race, hypertension, hyperlipidemia, obesity, smoking, CMT and axial length. The number of females decreased as the severity progressed (*p* = 0.003). A significant difference in hemoglobin A1c (HbA1c) was found among the four groups (*p* < 0.001): the DM without DR group (7.8 ± 2.0%) was significantly lower than the NPDR group (9.4 ± 2.3%, *p* = 0.001) and the PDR group (10.2 ± 2.8%, *p* < 0.001); there was no significant difference between the NPDR group and the PDR group. No significant difference of insulin status was found among the three DM groups. Detailed demographics are shown in Table 1.

**Table 1:** Demographic and clinical characteristics of study subjects.

	Total	No DM	DM without DR	NPDR	PDR	*p* value
Subjects	258	60	132	40	26	
Age (y)	52.11 ± 11.75	49.03 ± 1.82	53.51 ± 0.96	51.30 ± 1.72	53.35 ± 1.84	0.091
Sex—female, n (%)	124 (48)	30 (50)	72 (55)	15 (38)	7 (27)	0.033
Race/Ethnicity, n (%)						0.243
Non-Hispanic White	30 (12)	9 (15)	12 (9)	5 (12)	4 (15)	
Non-Hispanic Black	34 (13)	10 (17)	16 (12)	3 (8)	5 (19)	
Hispanic	122 (47)	29 (48)	63 (48)	17 (42)	13 (50)	
Asian	46 (18)	9 (15)	28 (21)	7 (18)	2 (8)	
Others	26 (10)	3 (5)	13 (10)	8 (20)	2 (8)	
HbA1c (%)	8.4 ± 2.3	N/A	7.8 ± 1.8	9.4 ± 0.4	10.2 ± 0.6	< 0.001
Hypertension, n (%)	133 (52)	30 (50)	69 (52)	21 (53)	13 (50)	0.981
hyperlipidemia, n (%)	144 (56)	32 (53)	70 (53)	25 (63)	13 (50)	0.740
Obesity, n (%)	110 (43)	27 (45)	57 (43)	17 (43)	9 (35)	0.783
Insulin status, n (%)	48 (19)	N/A	32 (24)	8 (20)	8 (31)	0.608
Smoking history, n (%)	102 (40)	18 (30)	54 (41)	19 (48)	11 (42)	0.311
Axial length (mm) *	23.55 ± 0.96	23.67 ± 0.14	23.50 ± 0.09	23.59 ± 0.15	23.33 ± 0.32	0.595
CMT (μm) *	239 ± 20	234 ± 16	238 ± 21	244 ± 18	241 ± 25	0.100
SS	9.08 ± 0.52	9.07 ± 0.45	9.14 ± 0.54	9.05 ± 0.50	8.88 ± 0.59	0.144

DM, diabetic retinopathy; DR, diabetic retinopathy; NPDR, non-proliferative diabetic retinopathy; PDR, proliferative diabetic retinopathy; CMT, central macular thickness. SS, signal strength of OCTA images. * Axial length and CMT were calculated from the average value for both eyes of individual patient.

### Comparability of OCTA among groups

No significant media opacities preventing adequate imaging were present in any of the groups. The mean OCTA signal strength in the no DM, DM without DR, NPDR, and PDR groups were 9.1 ± 0.4, 9.1 ± 0.5, 9.1 ± 0.5 and 8.9 ± 0.6, respectively (*p* = 0.144).

### AIs in different groups

**Figure 1:**
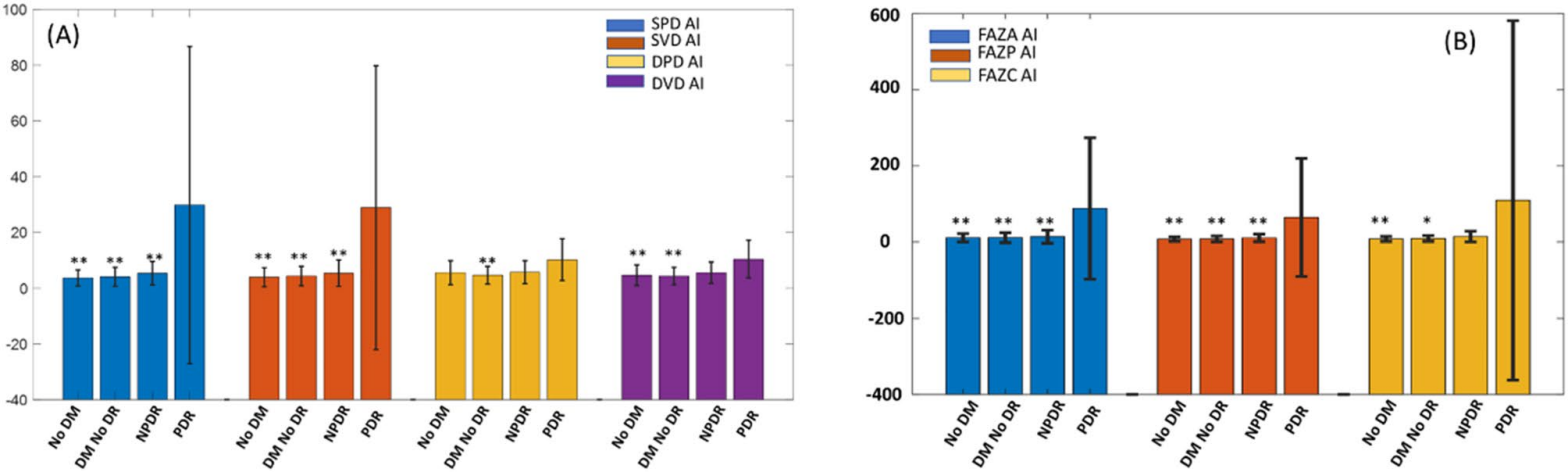
Comparison of AIs of Parameters Studied in This Study Among Four Groups: No DM, DM without DR, NPDR and PDR. (**A**) Parameters of vessel density from left to right: SPD, SVD, DPD and DVD. AIs of SPD and SVD in the PDR group were significantly larger than in the other three groups. AI of DPD in the PDR group was significantly larger than in the DM without DR group. AI of DVD in the PDR group was significantly larger than in the no DM group and the DM without DR group; (**B**) Parameters of FAZ from left to right: FAZA, FAZP and FAZC. AIs of FAZA and FAZP in the PDR group were significantly larger than in the other three groups. AI of FAZC in the PDR group was significantly larger than in the No DM and DM without DR groups. **p < 0.01 compared with the PDR group; *p < 0.05 compared with the PDR group. AI: asymmetry index; SPD: superficial perfusion density; SVD: superficial vessel density; DPD: deep perfusion density; DVD: deep vessel density; FAZA: foveal avascular zone area; FAZP: foveal avascular zone perimeter; FAZC: foveal avascular zone circularity.

AI of SPD was significantly higher in the PDR group (22.1 ± 26.0) compared with that of the no DM group (3.5 ± 2.6, *p* < 0.001), the DM without DR group (4.1 ± 3.4, *p* < 0.001) and the NPDR group (5.8 ± 4.4, *p* < 0.001). A similar result was found with AI of SVD, with higher SVD asymmetry in the PDR group (21.5 ± 24.0) than in the no DM group (3.8 ± 3.1, *p* < 0.001), the DM without DR group (4.4 ± 3.5, *p* < 0.001) and the NPDR group (5.8 ± 4.8, *p* < 0.001). The parameters of the deep microvasculature (DPD and DVD) had similar findings. Regarding the FAZ parameters, the asymmetry of FAZ area and perimeter were significantly larger in the PDR group than in the other three groups (Table 2, Fig. 1).

**Table 2:** Mean values and AIs of OCTA parameters between DR severity stage groups (%).

Group	No DM	DM without DR	NPDR	PDR	*p* value
AI of SPD	3.5 ± 2.6	4.1 ± 3.4	5.8 ± 4.4	22.1 ± 26.0	< 0.001
AI of SVD	3.8 ± 3.1	4.4 ± 3.5	5.8 ± 4.8	21.5 ± 24.0	< 0.001
AI of DPD	5.6 ± 4.1	4.8 ± 3.4	5.7 ± 4.1	10.5 ± 9.0	< 0.001
AI of DVD	4.5 ± 3.5	4.5 ± 3.3	5.5 ± 4.0	9.0 ± 6.6	< 0.001
AI of FAZA	10.9 ± 9.5	11.2 ± 10.6	13.2 ± 13.4	45.6 ± 41.0	< 0.001
AI of FAZP	7.6 ± 5.4	8.0 ± 7.1	10.5 ± 9.1	33.0 ± 35.3	< 0.001
AI of FAZC	8.1 ± 6.5	9.2 ± 7.6	14.7 ± 15.5	24.5 ± 36.2	< 0.001

The statistics were done by Kruskal–Wallis and Dunn's post hoc. *p* < 0.05 is considered to be a significant difference. AI: asymmetry index; SPD: superficial perfusion density; SVD: superficial vessel density; DPD: deep perfusion density; DVD: deep vessel density; FAZA: foveal avascular zone area; FAZP: foveal avascular zone perimeter; FAZC: foveal avascular zone circularity.

### Factors influencing AIs

Males tended to have greater asymmetry in FAZ area (29.81 ± 21.69 vs. 10.66 ± 9.85, *p* = 0.006), FAZ perimeter (5.87 ± 4.65 vs. 4.65 ± 3.59, *p* = 0.017), DVD (25.00 ± 83.75 vs. 13.58 ± 27.82, *p* = 0.033) and DPD (15.61 ± 71.26 vs. 12.75 ± 21.44; *p* = 0.015). In the multi-regression model adjusted by disease severity, sex only showed a significant relationship with the AI of DPD (beta = − 0.119 [95% CI: − 0.199, − 0.039], *p* = 0.05). No significant difference was seen in AI of SVD and SPD between males and females (*p* > 0.05). No significant difference in asymmetry of the studied parameters were found among the five races/ethnic groups.

Pearson’s correlation analyses showed that HbA1c was positively correlated with AI of SPD (R = 0.15, *p* = 0.036), AI of SVD (R = 0.15, *p* = 0.027), AI of FAZ perimeter (R = 0.20, *p* = 0.006) and AI of FAZ circularity (0.20, *p* = 0.007). Age did not show a significant relationship with any of the AI parameters. No correlation was observed between the mean axial length or the mean CMT and AI of all OCTA parameters. In the multi-regression model adjusted by age, sex, race, hypertension, hyperlipidemia, obesity, insulin status, and smoking history, HbA1c only showed a significant relationship with the AI of FAZ perimeter (beta = 0.007 [95% CI: 0.001, 0.013], *p* = 0.02) and AI of FAZ circularity (beta = 0.002 [95% CI: 0.000, 0.004], *p* = 0.022).

## Discussion

Many studies have identified OCTA changes in DR patients. For example, vessel density, and perfusion density^12,13^ progressively decrease with worsening DR severity; perfusion density and vessel density of the superficial layer have been correlated with the disease stage through linear regression analysis^14^; and DR progression is associated with enlarged FAZ and increased capillary nonperfusion area^15,16^. Given that it might take years from the time a diagnosis of DM is made until a vision complaint develops^17^, it is recognized that microvascular changes can take place ahead of the clinical diagnosis of NPDR. OCTA changes have also been found in mild NPDR and diabetic patients without DR, such as decreased vessel density of both the superficial and the deeper capillary plexus in type 2 diabetic patients with DR compared with healthy controls^18^, and reduced optic nerve head perfusion^19^ and increased extrafoveal avascular area^20^ in diabetic patients without a clinical diagnosis of DR.

In the present study, we report more pronounced inter-eye asymmetry in DR severity in patients with PDR, in all OCTA parameters. Diabetic patients without DR were likely to be more asymmetric than nondiabetic controls, but without a statistically significant difference. This indicates that diabetic microvasculature impairment could begin ahead of clinical signs and diagnosis, as has been previously reported, but these changes may be relatively symmetric in the early stages of the disease, whereas bilateral symmetry tends to be more affected as the DR severity advances. Jung et al. found an increasing quadrant asymmetry of capillary dropout based on a 3 mm x 3 mm averaged en-face OCTA in both the superficial and deep capillary plexus as DR severity increased. The amount of capillary dropout in the parafoveal macula may become more severe in the PDR eye compared to the fellow eye^21^.

By revealing that the microvascular changes of DR in the macula are not symmetric, the findings of this study could impact both research and clinical practice. For example, when designing research protocols that enroll one eye of each patient into a study, it may in some situations not be entirely accurate to select the laterality at random, because the chosen eye may not represent the two eyes collectively due to asymmetry. As for clinical practice, as OCTA imaging becomes more widely available, it could be helpful to incorporate this imaging on a routine basis for DR patients in order to detect differences between the two eyes, either to predict which eye will develop complications first or to allow more directed monitoring, possibly enabling eye laterality-driven precision medicine treatment in the future.

According to our study, the vessel density of the superficial capillary layer in the PDR group was significantly more asymmetric than in the NPDR group, while the difference of the deep capillary layer between the PDR group and the NPDR group was not significant, which indicates that the superficial capillary layer was more impacted in regard to bilateral symmetry. This could be unexpected in that it is believed that early diabetic vascular changes such as microaneurysm formation occur first and are more pronounced in the deep capillary plexus, as demonstrated by histopathology^22^ and OCTA^23^. However, there are inconsistencies in the literature, with some studies report that parameters derived from the superficial layer are more valuable for differentiating the stages of DR^24^, while others have supported deeper parameters^25^. While some of this variability in findings might result from differences in OCTA segmentation algorithms^26^, there have also been some results suggesting that superficial layers could be affected earlier than deep, while the deep plexus develops more severe changes as the disease progresses^27,28^. Given this background, one explanation of our finding could be that microvascular drop-out happens earlier than histopathologically recognized microaneurysms, and the superficial layer is more vulnerable to this alteration, and correspondingly the superficial capillary plexus more easily develops asymmetry between the two eyes. Another possibility is that the superficial data is more reliable than the deeper data, according to the limitations of the current OCTA imaging technique and the analysis software. It is known that analysis of the deeper capillary plexus is more likely to be confounded by projection artifacts and segmentation error^10,12^. The Cirrus device used in this study includes the removal of projection artifacts, and this particular methodology has been found to be superior to the systems applied in some other commercially available OCTA devices^29^. However, even with this correction applied to the images, subtle differences in the deep layers could be impacted by such artifacts, masking symmetry differences between eyes. In addition, the vessel densities of the superficial and deeper layers were derived by two different methods, making their comparison less reliable. Therefore, it may be difficult to prove whether the apparently delayed onset of asymmetry in the deeper layer represents a real finding or a measurement artifact.

Enlargement and irregularity of the FAZ are well-known characteristics of DR. The present study's findings broaden our understanding of the impact of DR upon the FAZ: not only do FAZ enlargement and irregularity develop in each individual eye with the progression of DR, but also asymmetric enlargement and irregularity of the FAZ become more pronounced on a bilateral basis.

This study evaluated the impact of other factors upon DR asymmetry. A number of studies showed an increased tendency toward DR progression in men compared to women^30–35^. Several studies using OCTA have also reported sex-related differences in age-associated vessel density change, with men consistently showing greater decreases compared to women^36–38^. Sex may contribute independently to the development of asymmetry, with male sex being a determinant of greater asymmetry of perfusion density of DRL, in addition to DR progression. HbA1c was also found to be a risk factor for asymmetry of FAZ parameters. Similarly, previous studies proved that patients with higher HbA1c tended to have lower retinal vessel density and larger FAZ^39–41^. As such, our findings that both sex and HbA1c independently determine retinal vascular/FAZ asymmetry in DR are consistent with the principle that as vessel density declines, asymmetry is more likely to develop between the two eyes. However, age did not show significant correlation with the symmetry of OCTA parameters, although prior studies reported that both superficial and deep vessel density decrease and FAZA increases with increasing age^36–38^. If correct, this finding indicates that risk factors that increase DR severity may not all necessarily affect the symmetry of the two eyes.

This study has several limitations. Enrollment was not evenly matched among the groups, due to the relatively small sample size of the PDR group, and the relatively heterogenous population of groups might confound the results. In addition, the NPDR group was not divided into sub-groups (mild, moderate and severe NPDR), so progression of symmetry changes between those groups was not explored in this study. Third, one patient had different DR stages between the two eyes (one PDR while the other severe NPDR), which may add bias. However, the OCTA metrics in this single patient were not significantly different between the eyes, so this is unlikely to have affected the study’s results. Of note, allowing this single subject to be included in the study is consistent with the precedent of previous publications in the area of OCTA and diabetic retinopathy in which PDR and severe NPDR were combined into a single group^22,42^. Additionally, some segmentation errors might be unavoidable, limited by the current segmentation algorithm. Also, it is possible that the results could have been confounded by the fact that imaging is more difficult in patients with more advanced disease. Variations in signal strength can significantly impact the results of OCTA analysis^43^. However, we believe this did not impact the asymmetry assessment because there was no noticeable difference in signal strength between images from the various groups analyzed in this study. Carotid artery imaging was not performed, making it possible that OCTA asymmetry between eyes could have been influenced by differences in carotid flow between right and left. Finally, since the analysis was performed only on the central 3 x 3 mm area of the macula, this study cannot determine whether these asymmetry findings apply only in the macula or extend throughout the entire retinal vasculature.

## Materials and methods

### Patients

This retrospective study was approved by the Human Research Protection Program (HRPP) at the University of California, San Francisco (UCSF). The UCSF HRPP granted a waiver of consent, affirming that patient welfare would not be adversely affected by waiving informed consent. All research adhered to the tenets of the Declaration of Helsinki. Patients underwent ophthalmic imaging as part of the diabetic retinopathy screening program at Zuckerberg San Francisco General Hospital and Trauma Center between April and December 2018. All subjects had a history of DM and unknown retinopathy status at the time of screening. The department reading center graded the presence and severity of DR based upon ultra-widefield photographs, assigning eyes into three groups: DM without DR, non-proliferative DR (NPDR), and proliferative DR (PDR). When two eyes of the same patient were graded at different stages, a subject’s group was determined by the more affected eye. Similar aged subjects without diabetes who had undergone ophthalmic imaging in the course of usual care were referenced as a control group.

Exclusion criteria included: (1) patients with Type 1 DM; (2) any history of ocular injury, ocular surgery and other retinal-vitreous diseases except DR; (3) presence of diabetic macular edema or exudates; (4) DR patients with a treatment history of laser, intravitreal injection or vitrectomy; (5) low quality OCTA images: signal strength of 7 or less, or presence of significant motion artifacts, defocus or blur.

### Data collection

The demographics of all subjects including gender, age, and race, the most recent HbA1c, hypertension, hyperlipidemia, obesity and smoking status were recorded. Ultra-widefield fundus images (Optos Daytona, Optos Plc, Dunfermline, United Kingdom) were obtained for each patient. Axial length was measured with the IOL Master 700 (Carl Zeiss Meditec, Dublin, CA, USA). OCTA was performed using a Cirrus high-definition–OCT instrument (Model 5000, Carl Zeiss Meditec) with a scan of 3 × 3 mm square area centered at the fovea after pupillary dilation with mydriatic eyedrops. Both eyes of each participant were imaged with a scan comprising 245 clusters of B-scans repeated four times, in which each B-scan consisted of 245 A-scans. The effect of eye motion-related artifacts was minimized by the use of FastTrac eye tracking software. Only images with a signal strength greater than 7, minimal motion artifacts, decentration from the foveal center of less than 20 microns, and minimal evidence of obscuration by media opacities were considered for analysis.

According to Cirrus segmentation algorithms, the inner retina, identified as the layer between the inner limiting membrane and an offset 110 μm from the retinal pigment epithelium layer, was further divided into the superficial retinal layer (SRL, superficial 70%) and deeper retinal layer (DRL, the deeper 30% remaining). Every examination was checked manually to ensure the right segmentation. The vessel density (SVD) and perfusion density (SPD) of the SRL as well as central macular thickness (CMT) were generated automatically in the Cirrus analysis software. Foveal avascular zone was also identified automatically in Cirrus, followed by checking and adjusting manually. FAZ area (FAZA), perimeter (FAZP) and circularity (FAZC) were generated subsequently.

Circularity was calculated with the formula:^44^

$${4}\pi \; \times \;{\text{FAZA}}/\left( {{\text{FAZP}}} \right).$$

The images of the DRL were analyzed with ImageJ software (1.8.0_112, http://imagej.nih.gov/ij/; National Institutes of Health, Bethesda, Maryland, USA) to acquire deep vessel density (DVD) and deep perfusion density (DPD). DPD was calculated as the percentage of pixels occupied by blood vessels in the binary image of the grayscale OCTA image. DVD was defined as the ratio of skeletonized vessel length to the total area with a skeletonized image. Superficial and deep vessel density and parameters of FAZ were corrected for axial length following the method reported previously^45^.

The asymmetry index (AI), calculated via the formula:

$${\text{AI}} = {\text{abs}}\left( {{\text{OD}} - {\text{OS}}} \right)/({\text{OD}}\, + \,{\text{OS}})*{2}*{1}00,$$

(unit: %, OD was the measurement of right eye and OS was the measurement of left eye), was used to assess the asymmetry of the parameters above, as was described in a previous study^46^.

### Statistical analysis

Statistical analyses were conducted using IBM SPSS (Version 22.0, Chicago, IL, USA). Age, HbA1c, CMT, axial length and AIs were presented as means ± standard deviations (SDs). The data distribution was examined using the Kolmogorov–Smirnov test. The intergroup difference of distribution of gender and race was tested via Chi-Squared Test. Fisher’s exact test was used when the table consisted of a cell where the expected number of frequencies was fewer than 5. Tests of homogeneity of variances were done before inter-group comparison. One-way ANOVA and the Dunn-Bonferroni test were used to evaluate inter-group differences. If the variances were of heterogeneity, a Kruskal–Wallis and Dunn's post hoc were used. The correlations between AIs and age, HbA1c, were analyzed via Pearson correlation tests. We also used a multi-regression model adjusted by age, sex, race, hypertension, hyperlipidemia obesity, DR grade, insulin status, and smoking history, to analyze the relationship between AIs and HbA1c. Statistical significance was defined as *p* < 0.05 (two tailed).

### Ethics approval and consent to participate

Institutional review board approval was obtained from the UCSF Human Research Protection Program, #17–21703. A waiver of consent to participate was obtained since the study was a retrospective review.

## Conclusions

This study provides insight into the interocular symmetry patterns of DR, based on retinal microvascular changes revealed by OCTA. We found that the two eyes of PDR patients were significantly asymmetric in both vascular density and FAZ metrics. In addition to DR severity, HbA1c and sex were risk factors that influenced symmetry. The study suggests that special attention to right-left asymmetry in DR is warranted in both research and clinical practice.

## Supplementary Information

Supplementary Information.

## Data Availability

The dataset generated during the current study is included with this report as supplemental material.
